# Clinical and Radiological Features of Menetrier’s Disease: A Case Report and Review of the Literature

**DOI:** 10.7759/cureus.45537

**Published:** 2023-09-19

**Authors:** Myles Keener, Andrew Waack, Meghana Ranabothu, Akash Ranabothu, Venkatramana R Vattipally

**Affiliations:** 1 College of Medicine and Life Sciences, The University of Toledo College of Medicine and Life Sciences, Toledo, USA; 2 Neurological Surgery, The University of Toledo College of Medicine and Life Sciences, Toledo, USA; 3 College of Liberal Arts and Sciences, Grand Valley State University, Allendale, USA; 4 Radiology, Advanced Radiology Services, P.C., Grand Rapids, USA

**Keywords:** esophagogastroduodenoscopy, gastrointestinal (gi), tgf-a, cytomegalovirus (cmv), menetrier's disease, helicobacter pylori

## Abstract

We present a case report describing the diagnosis and management of a patient who presents with a rare diagnosis of Menetrier’s disease. This condition poses a diagnostic challenge to clinicians due to its nonspecific clinical presentation and is oftentimes misdiagnosed for more common gastric disorders. Menetrier’s disease is characterized by gastric mucosal hypertrophy and subsequent protein loss, resulting in gastric symptoms and widespread edema. While the etiology remains unclear, notable associations have been observed with* Helicobacter pylori *(*H. pylori*) infection and overexpression of transforming growth factor-alpha (TGF-a). The management often involves supportive measures with medical and surgical interventions for refractory cases and when necessary. This report includes a comprehensive review of the literature on the clinical presentation, diagnostic approach, and management of this rare disease. By documenting such cases in the medical literature, we aim to enhance the clinician’s ability to recognize and manage this disorder, thereby preventing the development of more severe manifestations such as gastric carcinoma.

## Introduction

Menetrier’s disease is a rare disorder characterized by excessive gastric mucosal hypertrophy with associated protein loss. Currently, there have been fewer than 1000 reported cases of this disease [1]. The clinical presentation is often nonspecific and can resemble signs and symptoms of gastritis. However, the protein loss can cause widespread edema, providing a clearer picture of the diagnosis of Menetrier. A detrimental complication of Menetrier’s disease is the evolution to gastric adenocarcinoma. Because of the rarity of the disease and the often vague clinical presentation, it is important for clinicians to conduct a comprehensive diagnostic workup to prevent the development of further harm to the patient.

## Case presentation

A 61-year-old female with a prior medical history of pre-diabetes, anemia, gastroesophageal reflux disease (GERD), positive smoking history, and no significant family history underwent a routine low-dose computed tomographic (LDCT) lung cancer screening. LDCT revealed bilateral pulmonary nodules and incidentally detected a moderate hiatal hernia with a patulous esophagus.

A routine esophagogastroduodenoscopy (EGD) for hiatal hernia repair was later performed. Inspection of the stomach revealed a moderately sized hiatal hernia and a large mass-like mucosal irregularity with polypoid areas extending from the gastroesophageal junction to the antrum along the greater curvature of the stomach. Extensive biopsies were obtained, and the operating surgeon elected to postpone the hernia repair for further workup.

Pathological analysis of the surgical specimens revealed multiple fundic gland polyps with negative immunostaining for *Helicobacter pylori* (*H. pylori*). Some of the polyps exhibited reactive epithelial changes in the deep glands without any evidence of malignancy.

**Figure 1:**
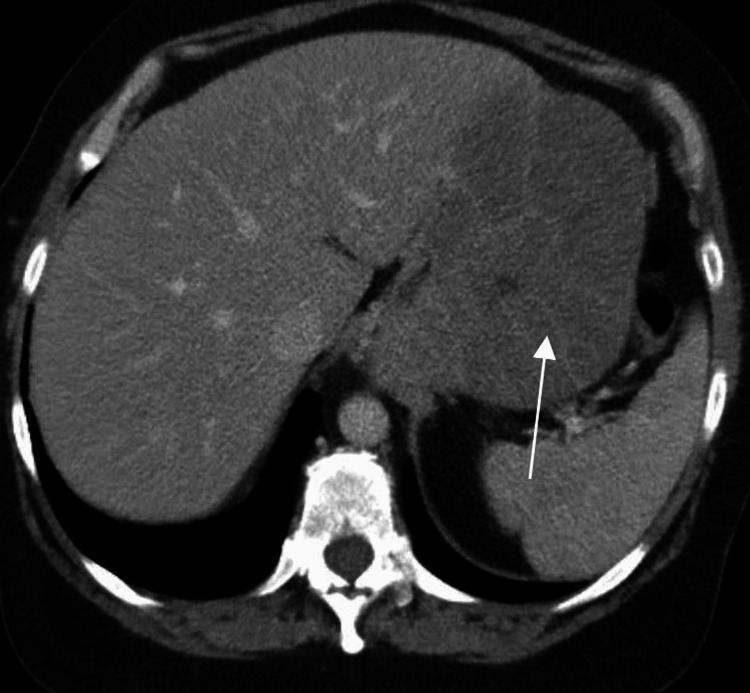
Axial IV contrast-enhanced CT image demonstrates marked rugal fold hypertrophy (white arrow)

**Figure 2:**
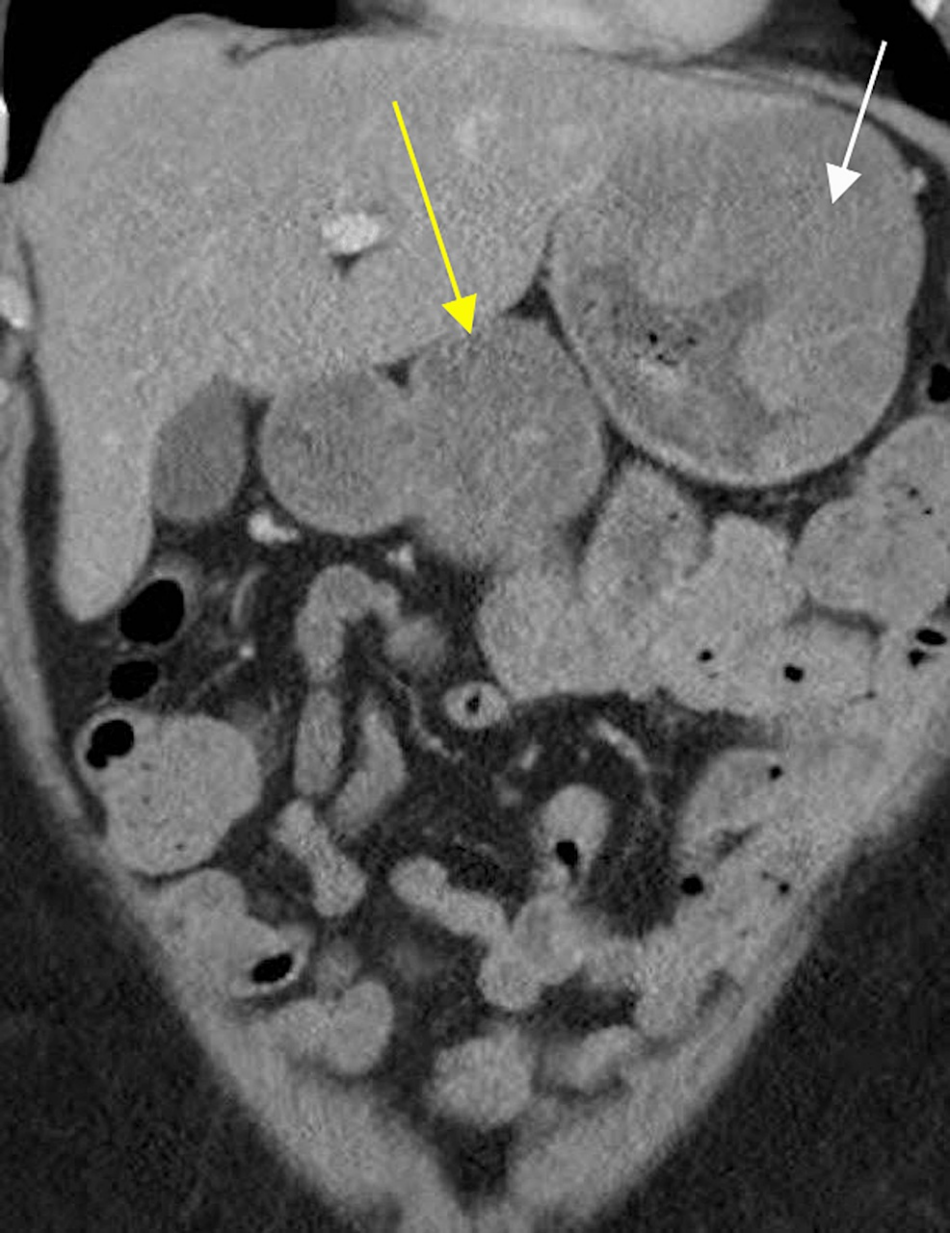
Coronal IV contrast-enhanced CT image demonstrates marked gastric wall thickening secondary to extensive rugal fold hypertrophy (white arrow). The antrum does not demonstrate wall thickening (yellow arrow).

**Figure 3:**
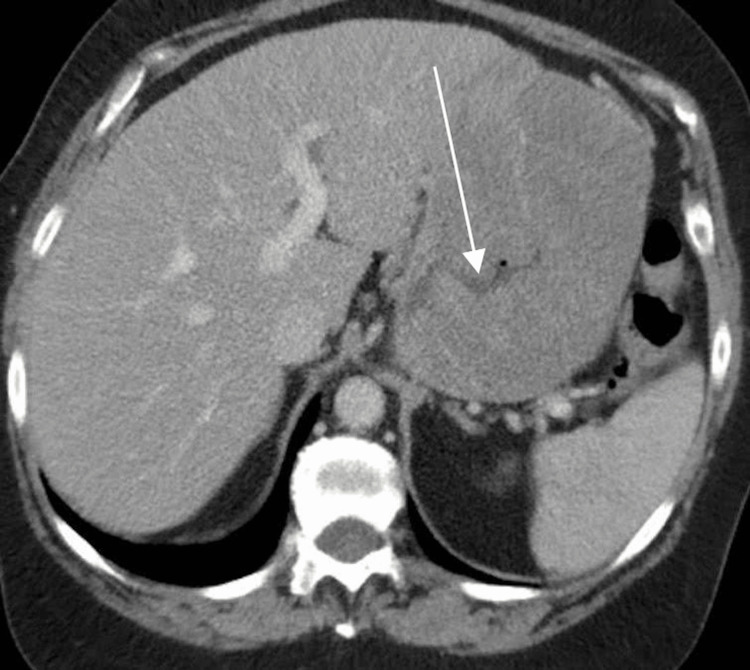
Axial IV contrast-enhanced CT image demonstrates marked rugal fold hypertrophy causing lumen narrowing (white arrow)

**Figure 4:**
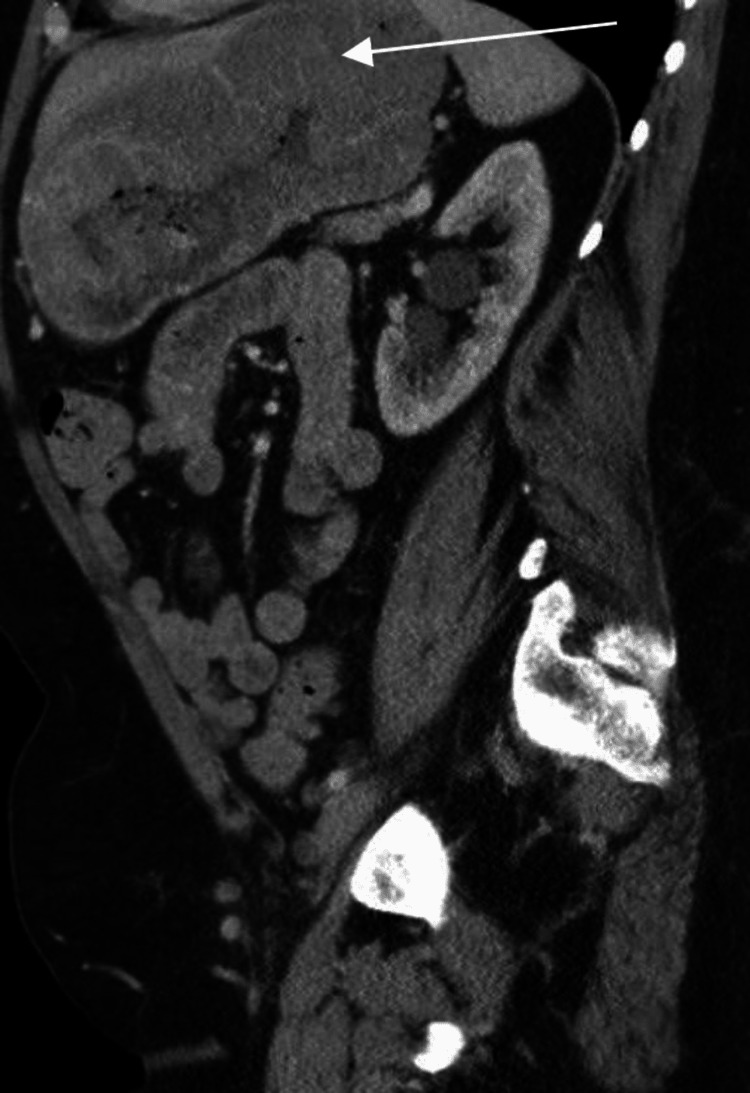
Sagittal IV contrast-enhanced CT image demonstrates marked gastric wall hypertrophy (white arrow)

Follow-up contrast-enhanced computed tomography (CT) chest, abdomen, and pelvis was performed two weeks later (Figures 1-4). CT confirmed a moderate size hiatal hernia and demonstrated eccentric thickening and enhancement of the wall of the fundus and greater curvature of the stomach, creating a "cerebriform" appearance. Even thicker, diffuse heterogeneous mass-like mucosal thickening was seen throughout the visualized gastric cardia and along the distal gastric body along the greater curvature. Of note, there was sparing of the antrum.

**Figure 5:**
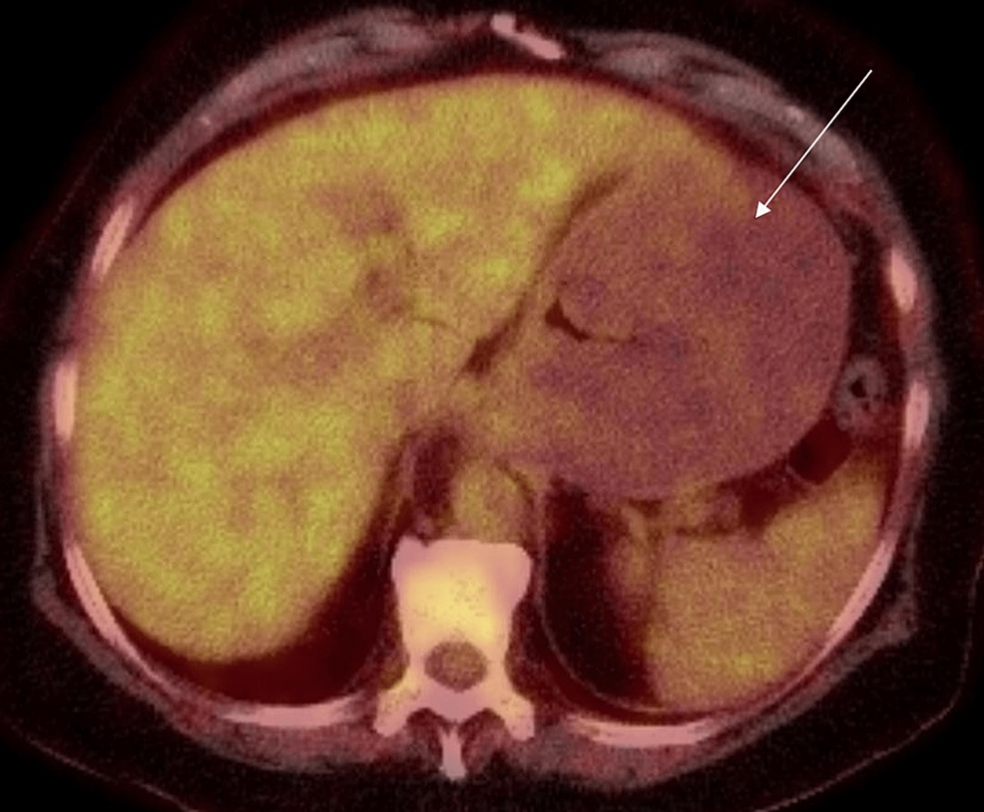
Axial view PET-CT demonstrating non-hypermetabolic gastric wall thickening (white arrow)

Subsequent positron emission tomography-CT (PET-CT) of the skull base to mid-thigh was performed two weeks later (Figure 5), which demonstrated moderate-sized hiatal hernia with marked thickening of the wall of the fundus and body of the stomach with sparing of the lesser curvature and antrum. PET-CT did not demonstrate increased metabolic activity within the stomach.

The patient's significant labs included a low total protein (6.2 g/dL) and albumin (3.1 g/dL). Collectively, imaging, labs, and pathologic findings suggested a diagnosis of Menetrier's disease. The decision was made to treat the patient symptomatically with proton pump inhibitor therapy and to not proceed with surgery for the hiatal hernia. The patient consented to undergo surveillance endoscopy every two to three years due to the risk of future development of gastric cancer.

## Discussion

Menetrier's disease is a rare gastric disorder that causes enlargement of gastric mucus-secreting cells with subsequent atrophy of the acid-secreting glands. It is not well-documented, with fewer than 1000 cases currently reported in the literature [1]. The disease tends to affect males slightly more than females and typically presents between the ages of 30 and 60 years with an average age of 55 [1,2]. 

The etiology of Menetrier’s disease is currently not fully understood. Interestingly, there is a notable association with *H. pylori* infection [3,4]. *H. pylori* can cause the development of gastritis and peptic ulcer disease. However, it is not known whether this leads to Menetrier's or if it is an unrelated, coincidental association. One study reported that *H. pylori* was present in 90% of Menetrier cases [4]. There is also a strong correlation with the overexpression of transforming growth factor-alpha (TGF-a), an epidermal growth factor receptor (EGFR) that is thought to play a major role in the proliferation of the gastric epithelium and gastric mucosal cells [5]. It is theorized that *H. pylori *plays a role in this overexpression. Although *H. pylori* has the most notable association with Menetrier's disease, there have been other less-known associations with infections such as CMV, herpes simplex virus, *Giardia lamblia*, and mycoplasma pneumonia [6,7]. There are rare cases in families that may suggest a weak genetic component, with one family having a mutation in the SMAD4 gene [1]. However, it remains that Menetrier is most likely an acquired disease rather than an inherited one.

The hyperplasia and hypertrophy of the gastric body cause a notable, enlarged, and rugated appearance of the gastric lining. The imbalance between mucus production and acid secretion due to the hypertrophy of mucus-secreting glands and epithelium results in impaired protein absorption. This protein loss has several manifestations, specifically with the loss of albumin and other carrier molecules the body depends on for transport.

The clinical presentation of Menetrier's disease is often nonspecific and can be misinterpreted as other more common abdominal diseases and disorders. Most individuals present with upper abdominal pain with and typically without nausea and vomiting. Often, the patients note early satiety, most likely attributed to the increased gastric surface area. The protein loss, specifically albumin, causes a state of fluid extravasation in peripheral tissues, leading to edema, most prominent in the lower extremities. Patients can also present with gastrointestinal bleeding [5]. There have been few reports of Menetrier presenting with intussusception, causing small bowel obstruction [1,2]. Although the disease is rarer in children, the presentation in this population often follows a transient viral illness [6].

The diagnostic workup for Menetrier’s disease often begins with a physical examination, medical history assessment, and basic laboratory tests such as complete blood count (CBC) and comprehensive metabolic panel (CMP). When protein levels, particularly albumin, are low, further imaging studies should be conducted to investigate protein-losing gastropathy. It is also recommended to screen for active *H. pylori* and cytomegalovirus (CMV) infection [8].

Imaging studies can be of many, including a radiological swallow with either barium or water-soluble contrast, allowing the radiologist to visualize the gastrointestinal tract and detect any abnormalities in size and shape. An esophagogastroduodenoscopy (EGD) is widely used in gastrointestinal (GI) practice to grasp a better and more direct view of the gastrointestinal lining in both the esophagus and the stomach. During EGD, gastroenterologists can assess hypertrophied gastric lining and folds. In the classic form of this disease, the hypertrophy is typically localized to the fundus with sparing of the antrum [2]. The criterion for an enlarged gastric fold is that which measures greater than 1.0 cm by radiologic studies [9]. CT scans and MRI can be used to evaluate gastric wall thickness as well as detect any associated complications that can arise with Menetrier such as gastric ulcers and even gastric carcinoma. These images can also identify lymphadenopathy, which can be a nonspecific finding or an early indication of carcinoma development.

Biopsies are also taken with EGD, allowing the pathologist to examine the tissue under the microscope, aiding in the diagnosis. Biopsy studies reveal gastric fundic mucosa hyperplasia and dilation of the fundic glands, as well as inflammatory cell infiltration at the lamina propria [5,6,10].

The management of Menetrier’s disease is largely supportive with a focus on nutritional replenishment. Patients are advised to follow a high-protein diet with frequent electrolyte monitoring and supplementation as needed. As mentioned previously, there is an association between *H. pylori *infection and Menetrier’s disease. Therefore, most patients are given antibiotics and proton pump inhibitor therapy for eradication and symptom relief. Steroids can be used to decrease inflammation and reduce gastric hypertrophy. Patients can also be given cetuximab and long-acting octreotide [5,11]. Cetuximab is a monoclonal antibody against immunoglobulin G1 (IgG1), which inhibits the binding of TGF-a to the EGF receptor. In a case series, there was a significant improvement in patient response to cetuximab [12]. If young or immunocompromised, Ganciclovir can be considered [6]. In patients whose disease is refractory to medical management or in patients with a risk of clinical deterioration, surgical intervention is utilized. If the disease is localized to the antrum, partial gastric resection can be done; however, if it is widespread, total gastrectomy is performed [3,13]. Surgical intervention also reduces the risk of gastric cancer development.

Menetrier’s disease is a chronic condition with rare spontaneous regression and remission. Patient response to symptomatic and supportive treatment varies. Some cases have reported complete symptom resolution and reduction in gastric hypertrophy with the eradication of *H. pylori* infection [14]. The overall risk of developing gastric adenocarcinoma is relatively low, estimated at 8.9% [15]. Although this risk is low, frequent monitoring with annual endoscopies and biopsies is recommended [16]. Unfortunately, there is no known cure for Menetrier’s disease, underscoring the importance of close patient follow-up to monitor the disease and prevent complications.

## Conclusions

Menetrier's disease is a rare disorder that should be suspected in patients with upper GI complaints and hypertrophied gastric mucosa. With a rather broad differential diagnosis consisting of Zollinger-Ellison syndrome, hypertrophic lymphocytic gastritis, hypertrophic hypersecretory gastropathy, gastric adenocarcinoma, gastric polyps, infections such as histoplasmosis and tuberculosis, autoimmune-like inflammatory conditions such as sarcoidosis, and more commonly, gastrointestinal disease, it is often overlooked in the diagnostic workup. Therefore, it is crucial for clinicians to conduct a thorough evaluation and maintain a high clinical suspicion when there is concurrent *H. pylori *infection and/or imaging findings suggestive of hypertrophied gastric mucosa to avoid missing this disease.
